# High Expression of the Tumor Suppressor Protein ITIH5 in Cholangiocarcinomas Correlates with a Favorable Prognosis

**DOI:** 10.3390/cancers16213647

**Published:** 2024-10-29

**Authors:** Verena J. Dreyer, Jia-Xin Shi, Michael Rose, Maureen T. Onyuro, Florian Steib, Lars Hilgers, Lancelot Seillier, Jana Dietrich, Janik Riese, Steffen K. Meurer, Ralf Weiskirchen, Ulf Neumann, Lara Heij, Tom Luedde, Sven H. Loosen, Isabella Lurje, Georg Lurje, Nadine T. Gaisa, Danny Jonigk, Jan Bednarsch, Edgar Dahl, Nadina Ortiz Brüchle

**Affiliations:** 1 Institute of Pathology, Medical Faculty, RWTH Aachen University, 52074 Aachen, Germany; verena.dreyer@rwth-aachen.de (V.J.D.); jishi@ukaachen.de (J.-X.S.); mrose@ukaachen.de (M.R.); maureen.onyuro@yahoo.de (M.T.O.); florian.steib@patho-trier.de (F.S.); lars.hilgers@rwth-aachen.de (L.H.); lseillier@ukaachen.de (L.S.); jdietrich@ukaachen.de (J.D.); jariese@ukaachen.de (J.R.); nadine.gaisa@uniklinikulm.de (N.T.G.); djonigk@ukaachen.de (D.J.); nortiz-bruechle@ukaachen.de (N.O.B.); 2 Center for Integrated Oncology Aachen Bonn Cologne Düsseldorf (CIO ABCD), 52074 Aachen, Germany; 3 Institute of Pathology, University Hospital, University of Ulm, 89081 Ulm, Germany; 4 Institute of Molecular Pathobiochemistry, Experimental Gene Therapy and Clinical Chemistry (IFMPEGKC), RWTH University Hospital Aachen, 52074 Aachen, Germany; smeurer@ukaachen.de (S.K.M.); rweiskirchen@ukaachen.de (R.W.); 5 Department of Surgery and Transplantation, University Hospital Essen, 45147 Essen, Germany; ulf.neumann@uk-essen.de (U.N.); lheij@ukaachen.de (L.H.); jan.bednarsch@uk-essen.de (J.B.); 6 Department of Pathology, University Hospital Essen, 45147 Essen, Germany; 7 Department of Renal and Hypertensive Disorders, Rheumatological and Immunological Diseases (Medical Clinic II), Medical Faculty, RWTH Aachen University, 52074 Aachen, Germany; 8 Department of Pathology and Clinical Bioinformatics, Erasmus University Medical Centre, 3015 CN Rotterdam, The Netherlands; 9 Department of Gastroenterology, Hepatology and Infectious Diseases, Heinrich Heine University Düsseldorf, 40225 Düsseldorf, Germany; tom.luedde@med.uni-duesseldorf.de (T.L.); sven.loosen@med.uni-duesseldorf.de (S.H.L.); 10 Department of Hepatology and Gastroenterology, Campus Charité Mitte and Campus Virchow-Klinikum, Charité-Universitätsmedizin Berlin, 10117 Berlin, Germany; isabella.lurje@charite.de; 11 Department of General, Visceral and Transplantation Surgery, Heidelberg University Hospital, 69120 Heidelberg, Germany; georg.lurje@med.uniheidelberg.de; 12 German Center for Lung Research (DZL), BREATH, 30625 Hanover, Germany

**Keywords:** Cholangiocarcinoma (CCA), bile duct cancer, ITIH5, class II tumor suppressor gene (C2TSG), ITIH5 upregulation

## Abstract

**Background**: Cholangiocarcinoma (CCA) are aggressive bile duct cancers with a poor prognosis for which there are only few established prognostic biomarkers and molecular targets available. The gene *ITIH5*, a known class II tumor suppressor gene (C2TSG), encodes a secreted protein of the extracellular matrix mediating tumor suppressive properties. Recently, it was surprisingly found that the ITIH5 protein is specifically upregulated in CCAs and that ITIH5 detection in blood could be an excellent liquid biopsy marker for indicating the presence of a CCA tumor in a patient. We therefore investigated whether patients with CCAs with abundant versus low ITIH5 protein expression also differ in their prognosis. **Methods**: To clarify this question, a large CCA cohort (n = 175) was examined using immunohistochemistry on a tissue microarray (TMA). **Results**: Abundant ITIH5 expression in CCA was associated with favorable survival, a low UICC stage and the absence of perineural invasion (PNI). **Conclusions**: ITIH5 has biomarker potential not only for the early detection of CCA from blood-based liquid biopsies but also as a prognostic tissue biomarker for risk stratification. Our results suggest that the upregulation of ITIH5 is particularly abundant in intrahepatic CCAs (iCCA). The mechanisms mediating the strong initial upregulation of ITIH5 during the oncogenic transformation of bile duct cells are still unclear.

## Simple Summary

*Inter-alpha-trypsin inhibitor-5* (*ITIH5*), a class II tumor suppressor gene, encodes a protein that is lost during tumor progression in many solid cancers. Unexpectedly, however, ITIH5 is significantly upregulated in cholangiocarcinoma (CCA), making it a potential liquid biopsy marker for early CCA detection, as recently shown. Indeed, CCA is the first tumor entity described in which ITIH5 is upregulated rather than downregulated in the tumor compared to normal tissue. In our study, we demonstrate that CCAs with abundant ITIH5 protein expression have favorable survival, a low UICC stage and the absence of perineural invasion. The re-expression of ITIH5 impairs the colony growth of cholangiocarcinoma cell lines. Although ITIH5 is upregulated in the tumor, it retains its tumor-suppressive function in CCA, similar to other tumor entities such as breast cancer and pancreatic cancer, where it is downregulated. Thus, ITIH5 may have both diagnostic and prognostic biomarker potential in CCA. The mechanisms of ITIH5 upregulation, particularly in intrahepatic CCAs, during oncogenic transformation remain unclear, but their elucidation could improve future CCA therapies.

## 1. Introduction

Cholangiocarcinoma (CCA) is a malignant tumor of the bile duct system and the second most common primary liver tumor [1,2]. CCAs are classified as intrahepatic (iCCA) or extrahepatic (eCCA) according to their anatomical location. eCCAs are located either perihilar (pCCA) near the bifurcation of the common hepatic duct and the cystic duct or distal (dCCA) to the liver [3]. Gallbladder carcinomas are considered separately [4]. In addition to anatomical location, CCA subtypes also differ in epidemiology (with the highest incidence in Southeast Asia [5]), immunological status [6], genome aberrations [7], etiopathogenesis and treatment [3,8,9]. Risk factors include primary sclerosing cholangitis [10], hepatolithiasis, biliary malformations and hepatobiliary parasites [11]. Since most patients remain asymptomatic in the early tumor stages and the occurring symptoms are nonspecific, CCAs are usually only diagnosed in advanced stages [8,12]. The gold standard for therapy is surgical resection or, if the tumors are not resectable, chemotherapy with gemcitabine and cisplatin [13]. However, the prognosis is still very poor, even in complete resections (R0) [14].

The gene encoding Inter-alpha-trypsin inhibitor heavy chain 5 (ITIH5) was first described in 2004 [15]. *ITIH5* is located on human chromosome 10p14 and contains 14 exons. The ITIH5 protein harbors the typical domains found in the ITI protein family, i.e., the VIT domain (Interpro number IPR013694) and the vWA domain (Interpro number SM00327), as well as a conserved cleavage site [16]. In healthy tissues, including placenta, breast and ovaries, abundant ITIH5 expression has been found in epithelial cells [15]. *ITIH5* has been identified as an epigenetically silenced (class II) tumor suppressor gene (which we abbreviate as C2TSG [17]) in breast cancer [18] and multiple other tumor entities—for example, bladder [19] and colon cancer. In addition, *ITIH5* may act as a metastasis suppressor gene in several tumor entities [20]; this property has been best studied in pancreatic cancer [21,22,23,24] and breast cancer [18]. In all cancer tissues analyzed so far, the downregulation or loss of ITIH5 was shown to be caused by promoter DNA hypermethylation and was associated with unfavorable prognosis in most cases [16,18,19,22,25,26]. In cancer cell models, the forced overexpression of ITIH5 often slows down cellular characteristics that constitute the hallmarks of cancer [27], i.e., triggering a reduction in cell proliferation, migration and invasion [16,19,21,25]. While the functions of the individual domains of ITIH5 have not been fully elucidated, it is assumed that an important function of ITIH5 is the stabilization of the extracellular matrix (ECM). This is conveyed through the covalent binding of hyaluronic acid (HA), a ubiquitous component of the ECM [28]. The binding reaction occurs via the C-terminal amino acids in the conserved cleavage site of ITIH5 and is catalyzed by TSG-6, encoded by the *TNFAIP6* gene [18,29,30]. Young et al. showed that a modified ITIH5 protein, which lacks the signal peptide necessary for secretion, still has similar effects as the unmodified full-length ITIH5 protein in cellular models of pancreatic carcinoma, suggesting that ITIH5 may also act intracellularly [24]. Furthermore, Rose et al. showed recently that the VIT domain may play a central role in the tumor-suppressive function of ITIH5, as a shortened ITIH5 protein (161 amino acids), basically truncated to the VIT domain, still exhibits tumor-suppressive effects on various cancer cell lines [31].

Recently, it was found that the ITIH5 protein is specifically upregulated in CCAs and that the ITIH5 protein detected in blood serum could be a promising liquid biopsy marker for indicating the presence of a CCA in a patient [32]. In our study, we therefore investigated whether CCAs with abundant and low ITIH5 protein expression also differ in their prognosis. This is important since CCA is the first tumor entity described in which ITIH5 is upregulated rather than downregulated in the tumor compared to normal tissue. To clarify this question, a large CCA cohort with a total of 175 tumors was examined using ITIH5 immunohistochemistry on tissue microarrays (TMA). Remarkably, abundant ITIH5 expression was associated with favorable survival, indicating that ITIH5 continues to function as a tumor suppressor protein in CCA.

## 2. Materials and Methods

### 2.1. Human Bioprobes/Data Collection

The Cancer Genome Atlas (TCGA) dataset “CHOL” was accessed via UCSC Xena (https://xenabrowser.net/). The dataset contains mRNA expression data as normalized counts created with the Illumina HiSeq 2000 RNA Sequencing platform, DNA methylation data created with the Illumina Infinium HumanMethylation450 platform and corresponding clinical data. In the TCGA dataset “CHOL”, there are 36 CCA samples and 9 normal tissue samples, which match cases to 9 of the CCA samples taken from the same patients.

A normal tissue microarray (normal TMA) analyzed containing 27 samples of various normal tissues derived from intrahepatic bile ducts (n = 2), perihilar bile ducts (n = 6), distal bile ducts (n = 8), intrapancreatic bile ducts (n = 3) and gallbladder (n = 8) was stained and analyzed. This retrospective study was carried out according to the guidelines of the Declaration of Helsinki and approved by the local ethics committee of the Medical Faculty of RWTH Aachen University (ethical vote EK173/06 and EK 100/21).

The clinical data of the CCA tissue microarray (TMA) were obtained from RWTH Aachen University Hospital (ethical vote EK173/06). The CCA TMA contains 175 samples of intrahepatic (n = 93), perihilar (n = 79) CCA, mixed type (n = 2) and combined gall bladder tumors (n = 1). The additional gall bladder tumor sample was excluded from the analysis, as it has a different pathophysiology from CCA. For the Cox regression analysis, we excluded cases of death within one month post-surgery to more accurately assess the true impact of tumor biology on survival and to avoid the confounding effects of surgery-related mortality [33]. The clinical characteristics of the CCA TMA cohort and the TCGA cohort are shown in Tables S1 and S2.

For the methylation analysis of the *ITIH5* promoter in the TCGA dataset, six CpG sites, i.e., cg15924332, cg09445472, cg01382938, cg10119075, cg25575628 and cg10151473, were chosen based on the previous work of our group [16,21,25].

### 2.2. Cell Lines

The human extrahepatic cholangiocarcinoma cell lines EGI-1 (RRID: CVCL_1193) and CCC-5 (RRID: CVCL_LM83) were purchased from the DSMZ—German Collection of Microorganisms and Cell Cultures GmbH (Braunschweig, Germany). The intrahepatic cholangiocarcinoma cell line hCKC was a gift from the lab of Prof. Ralf Weiskirchen. All cell lines were authenticated within 12 months of being used in the study and were cultured as described previously [34] and regularly tested for the absence of mycoplasma infection using the PCR-based Venor^®^ GeM Mycoplasma Detection Kit (Minerva Biolabs, Berlin, Germany). The cells were cultured under standardized conditions (37 °C, 5 vol% CO_2_, 20% O_2_, 95% humidity). EGI-1 was cultivated in Gibco^TM^ Minimal Essential Medium (MEM), supplemented with 10% fetal bovine serum, 1 mM sodium pyruvate and Gibco^TM^ MEM amino acids (both essential and non-essential). CCC-5 was cultivated in Gibco^TM^ Dulbecco’s Modified Eagle Medium (DMEM) low glucose and Gibco Keratinocyte SFM at a 2:1 ratio, supplemented with 20% fetal bovine serum. Also, 50 U ml-1 penicillin, 50 mg ml-1 streptomycin and 2 mM L-glutamine were added to all media.

### 2.3. Immunohistochemistry

First, 2 µm slices were cut from the paraffin-embedded tissue with a microtome and transferred onto slides. For the automated immuno-staining, the Lab Vision Autostainer 360-2D (Thermo Fisher Scientific, Waltham, MA, USA) was used with the EnVision™ Flex detection kit (Agilent Dako, Santa Clara, CA, USA). The immunohistochemistry samples and TMAs were stained with a custom-made ITIH5 antibody (Pineda, Berlin, Germany) named “2013-Tier 3” in a dilution of 1:500. This ITIH5 antibody directed against the C-terminal end of the ITIH5 polypeptide has been described recently [35]. The secondary antibody was horseradish peroxidase (HRP)-conjugated, which oxidizes the chromogen 3,3′-Diaminobenzidine (DAB). For counter-staining and fixation hematoxylin, alcohol and xylene were used. ITIH5 expression was judged using the Immunoreactive score (IRS) [36]. The IRS score (0–12) is defined as the product of the staining intensity score (between 0 and 3) multiplied by the score of positive cells proportion (between 0 and 4).

### 2.4. Nucleic Acid Extraction and Reverse Transcription PCR

Nucleic acid extraction from cells was carried out using the NucleoSpin^®^ RNA plus kit (Macherey-Nagel, Düren, Germany) for RNA and the QIAamp DNA Mini Kit (QIAGEN, Hilden, Germany) for DNA according to the manufacturer’s instructions. The nucleic acid concentration was measured with the NanoDrop ND-1000 Spectrophotometer (VWR, Radnor, PA, USA). The isolated RNA was stored at −80 °C, and the DNA was stored at −20 °C. In total, 1 µg of the obtained RNA was used for reverse transcription with the Reverse Transcription System Kit (Promega, Madison, WI, USA).

### 2.5. Semiquantitative Real-Time PCR

For the semiquantitative PCR, the iTaq™ Universal SYBR^®^ Green One-Step Kit (Bio-Rad Laboratories, Munich, Germany) and the CFX96 Touch Real-time PCR Detection System (Bio-Rad Laboratories, Munich, Germany) were used. All reactions were performed in triplicate with the reference gene GAPDH. The primer sequences are listed in Table S3. The annealing temperatures were set to 60 °C. Melting curve analysis was conducted to control product specificity. The relative expression ratio of mRNAs in each group was calculated by the 2^−ΔΔCT^ method.

### 2.6. Pyrosequencing

First, 500 ng of DNA was bisulfite-converted for 16 h using the EZ DNA Methylation Kit (Zymo Research, Bad Homburg, Germany), following the manufacturer’s instructions. For Pyrosequencing, the PyroMark Q48 Autoprep Instrument (QIAGEN, Hilden, Germany) was used with the PyroMark PCR Kit, PyroMark Q48 Disc, Pyromark Q48 Magnetic Beads and PyroMark Q48 Advanced CpG Reagents (QIAGEN, Hilden, Germany). The primer sequences are shown in Supplementary Table S4.

### 2.7. In Vitro Demethylation

The whole-genome demethylation of human cholangiocarcinoma cell lines was performed as follows: The demethylation agent 5-aza-2′-deoxycytidine (DAC) was added to a final concentration of 5 μM on days 1, 2 and 3. On day 3, the cells were additionally treated with 300 nM trichostatin A (TSA) (Sigma-Aldrich, St. Louis, MO, USA). The cells were harvested on day 4 for RNA and DNA extraction.

### 2.8. Protein Isolation from Cells and Western Blot

SDS-polyacrylamide gel electrophoresis (SDS-page) and Western blotting were performed as previously described [37], with slight modifications. The extraction of proteins from the cells was performed with NuPage LDS sample buffer (Invitrogen Life Technologies, Darmstadt, Germany) containing dithiothreitol (DTT) for reducing conditions. The lysates were then sonicated using the Sonopuls HD2070 ultrasonic device (Bandelin electronic, Berlin, Germany). The protein lysates were separated in 4-12% Bis-Tris gels (Invitrogen Life Technologies, Darmstadt, Germany) using MOPS-SDS running buffer and then electroblotted onto nitrocellulose membranes (0.2 μm, GE Healthcare, Chicago, IL, USA). The antibodies used were the generated C-terminal ITIH5 antibody (1:1000, Pineda), which was previously described [19] with secondary HRP conjugated anti-rabbit (1:10,000, Dako-Agilent, Santa Clara, CA, USA). In addition, β-actin antibody (1:2000, Sigma-Aldrich, Deisenhofen, Germany) with the secondary HRP-conjugated anti-mouse antibody (1:10,000, Dako-Agilent, Santa Clara, CA, USA) was used to monitor equal protein loading. For antibody detection, the SuperSignal West Femto Maximum Sensitivity reagent (Thermo Fisher Scientific, Waltham, MA, USA) was used. The original Western blot images can be found in Supplemental Materials Figure S4.

### 2.9. Stable Transfection of EGI-1 and CCC-5 Cells

EGI-1 and CCC-5 cells were transfected with either the ITIH5-pBK-CMV vector, which contains full-length ITIH5, or with the mock vector [15]. FuGENE^®^ HD transfection reagent (Promega, Madison, WI, USA) was used for transfection according to the manufacturer’s instructions. An ideal ratio of transfection reagent to DNA of 3:1 was determined for both cell lines. 48 h after transfection, a cell dilution series was performed. From then on, the cells were cultivated in a conditioned medium treated with the antibiotic Geneticin (G418, Invitrogen Life Technologies, Darmstadt, Germany) as a selective agent for transfected clones at a concentration of 500 μg·mL^−1^ for EGI-1 and 1000 μg·mL^−1^ for CCC-5. After two to three weeks, single-cell colonies were picked and cultured. The mRNA and protein expression were analyzed by qPCR and Western blot. For the cell culture assays, four ITIH5 and four mock clones were selected for EGI-1, and two ITIH5 and two mock clones were selected for CCC-5.

### 2.10. XTT Assay

The XTT assay was performed with EGI-1 and CCC-5 ITIH5 and mock clones using the XTT Cell Proliferation Kit II (Roche Diagnostics, Rotkreuz, Switzerland), as previously described [26]. Three independent runs were performed.

### 2.11. CFA Assay

EGI-1, CCC-5 ITIH5 and mock clones were seeded in triplicate in six-well plates (EGI-1 500 cells per well, CCC-5 750 cells per well). The cells were cultivated under ideal conditions, and selection pressure by geneticin was maintained. After 14 days, the cells were fixated and stained with 3.5% formaldehyde, 80% methanol and 6.1 mM crystal violet for 30 min. Afterwards, the colonies were photographed with the Gel Jet Imager (Intas, Göttingen, Germany), and densitometric analysis was performed using ImageJ (National Institutes of Health, Bethesda, MD, USA). Three independent runs were performed.

### 2.12. Statistical Methods

The software used for statistical analysis was SPSS 27.0 (SPSS, Chicago, IL, USA) and GraphPad Prism 5.0 (GraphPad Software Inc., La Jolla, CA, USA). The nonparametric Mann–Whitney U test was applied to compare the two groups, and the nonparametric Kruskal–Wallis test was used to compare more than two groups. The survival analysis was performed with Kaplan–Meier curves and the log rank test. Univariate Cox regression was used to correlate clinicopathological features to survival. Multivariate Cox regression was performed for all clinicopathological features that showed a *p*-value of ≤0.5 in the univariate Cox regression in order to eliminate confounders. For correlation analysis, the Spearman’s rank correlation coefficient was used. Overall survival (OS) was measured from surgery until relapse (local/distant) and was censored for patients without evidence of tumor recurrence at the last follow-up date. Fisher’s exact test was used to analyze clinical pathological features. Differences were considered statistically significant if the two sides’ *p* values were less than 5% (<0.05). Scatter plots were illustrated as the Mean ± SEM.

## 3. Results

### 3.1. Expression of ITIH5 Is Strongly Upregulated in Cholangiocarcinoma with Special Emphasis in Intrahepatic Cholangiocarcinoma (iCCAs)

**Figure 1:**
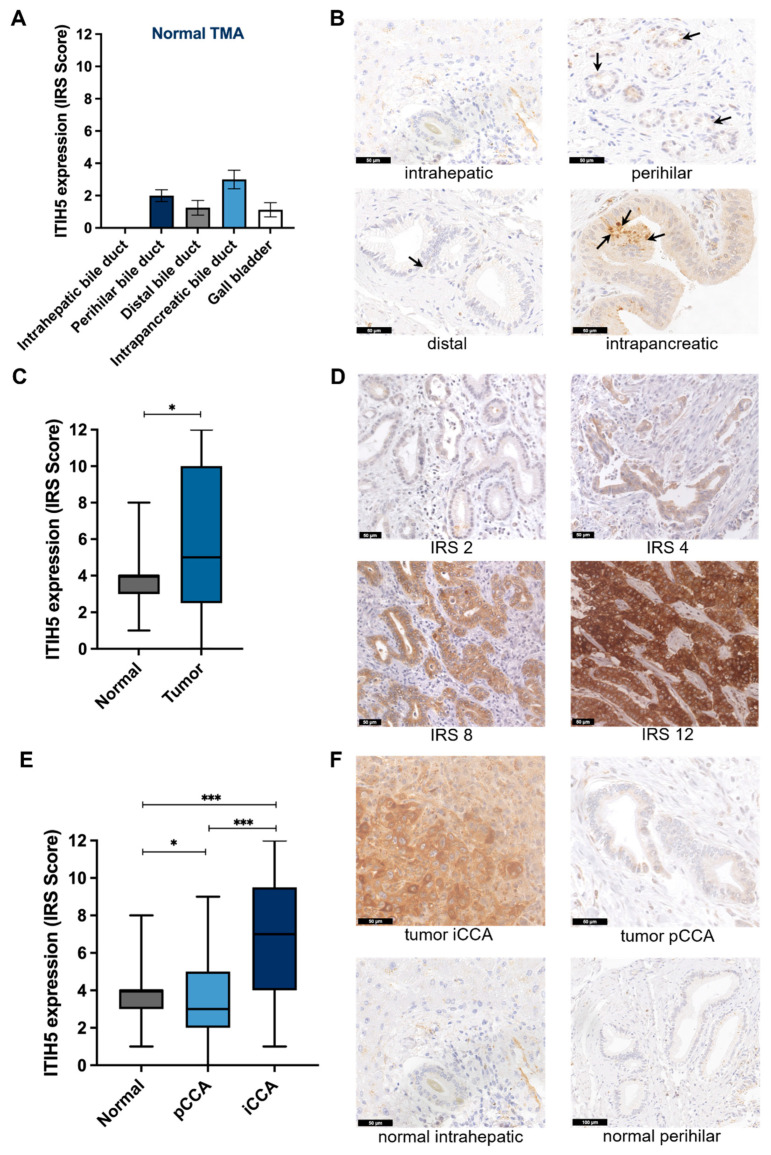
ITIH5 mRNA and protein expression is strongly upregulated in cholangiocarcinoma compared to normal tissue. (**A**) Low ITIH5 protein expression in different normal bile duct tissues, n = 27, mean ± SEM; (**B**) IHC images of normal tissue samples from different anatomical locations, stained with ITIH5 antibody. The arrows indicate sites with low ITIH5 expression in normal tissues; (**C**) mean ITIH5 protein expression in normal vs. tumor tissue in a CCA TMA, stained with ITIH5 antibody; quantification of protein expression by IRS score; (**D**) IHC images of tissues with different tumor ITIH5 protein expression levels, quantified by IRS score; (**E**) Mean ITIH5 protein expression in iCCA vs. pCCA from CCA TMA, with a significant upregulation of ITIH5 in iCCA; (**F**) IHC images of intrahepatic and perihilar tumor and normal tissue samples. * *p* < 0.05, *** *p* < 0.001.

First, the publicly available TCGA datasets for CCAs (n = 36) and corresponding normal tissue (n = 9) were analyzed. A significant upregulation (approx. 65-fold) of ITIH5 mRNA expression was detected in CCA tumor tissue compared to normal bile duct tissues (*p* < 0.0001 ***) (Figure S1). As the analysis of only nine normal tissues may fail to describe a representative pattern of ITIH5 expression, we analyzed ITIH5 protein expression in a larger collective of normal tissues (n = 27), which also reflected different areas of the bile duct. Figure 1A shows the median IRS scores of low ITIH5 expression (IRS 0 to 4) detectable in perihilar, distal and intrapancreatic bile duct tissue and the gallbladder, with minimal expression observed in intrahepatic bile duct tissue. Exemplary immunohistochemistry images of these topological distinct bile duct cells are shown in Figure 1B, showcasing the low ITIH5 expression in different anatomical locations of normal bile duct tissue. Subsequently, a comprehensive CCA cohort was examined, comprising 175 cholangiocarcinoma specimens arranged on a tissue microarray. Based on that, the upregulation of ITIH5 protein expression was significantly confirmed compared to normal tissue (mean IRS 3.99 in normal tissue vs. 5.43 in tumor tissue, *p* = 0.0159 *, Figure 1C). Figure 1D illustrates representative images of different IRS scores of ITIH5 expression in these CCA samples of the tissue microarray. Finally, sub-classifying the CCAs of the tissue microarray into subtypes (iCCA and pCCA) revealed that the ITIH5 protein is predominantly expressed in iCCAs compared to pCCAs (mean IRS 6.99 in iCCA vs. 3.52 in pCCA vs. 3.99 in normal tissue, *p* = 0.0124 *, *p* < 0.0001 ***, Figure 1E). Representative images of intrahepatic and perihilar tumors, along with normal tissue samples, are presented in Figure 1F, highlighting the elevated expression of ITIH5 in iCCAs.

There are only a few cholangiocarcinoma cell lines available worldwide, and these are even difficult to obtain. Therefore, we could only examine the mRNA expression and DNA methylation of ITIH5 in three CCA cell lines, i.e., EGI-1 and CCC-5 (extrahepatic cell lines) and hCKC (intrahepatic cell line). Indeed, the two extrahepatic cell lines showed very low ITIH5 expression, while the hCKC cell line showed moderate expression (Ct value of 28), and the two eCCA cell lines exhibited abundant promoter DNA methylation (Figure S2). The functional relationship between the methylation of the *ITIH5* promoter and the silencing of the *ITIH5* gene was further substantiated by *in vitro* demethylation experiments (Figure S3). For this purpose, the two cholangiocarcinoma cell lines CCC-5 and EGI-1 were treated with 5-aza-2′-deoxycytidine (AZA) or trichostatin A (TSA) or a combination of these two substances. AZA is a deoxycytidine analog that is typically used to activate methylated and silenced genes by promoter demethylation [38], while TSA is a well-characterized histone deacetylase (HDAC) inhibitor [39]. This treatment led to a significant restoration of ITIH5 mRNA expression (Figure S3). As reported in a previous work of our group, the upregulation of ITIH5 mRNA expression was particularly evident in the combination of the two epigenetic drugs [19]. Thus, this limited analysis of CCA cell lines reflects the picture observed in CCA tumors, i.e., lower ITIH5 mRNA expression in eCCA most likely caused by promoter DNA hypermethylation and considerably more abundant ITIH5 expression in iCCA.

### 3.2. Abundant ITIH5 Expression in CCA Is Associated with Favorable Overall Survival

**Figure 2:**
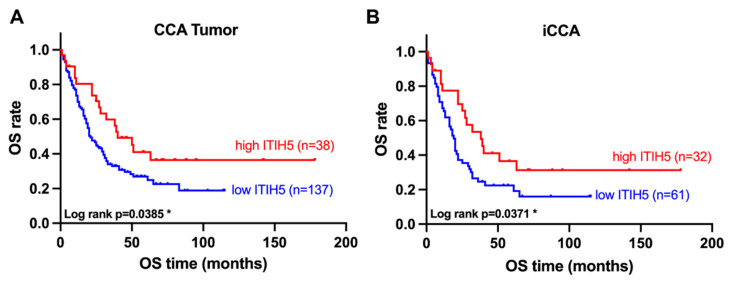
Survival analysis in terms of overall survival rate (OS). Statistical significance determined by log rank test. (**A**) Kaplan–Meier plot of CCA TMA data; ITIH5 expression grouped in IRS 0-8 (low and moderate ITIH5 expression, blue) and 8–12 (high ITIH5 expression, red); (**B**) Kaplan–Meier plot of CCA TMA, including only intrahepatic samples. * *p* < 0.05.

For the survival analysis of the tissue microarray CCA dataset, ITIH5 expression was dichotomized into low to moderate (IRS of 0 to 8) and high expression (IRS of 8 to 12) groups, as shown in Figure 2A. Patients with strong ITIH5 expression were characterized by a longer overall survival (log rank *p* = 0.0385 *). The median overall survival (OS) was nearly twice as long in the high ITIH5 expression group as in the low to moderate ITIH5 expression group (40 vs. 21 months). The risk of death was also significantly lower in the high ITIH5 expression group (HR = 0.5939, 95% CI = 0.3830–0.9209). In Figure 2B, an analysis was performed on only the intrahepatic cases of the tissue microarray. The observation of a survival benefit in the group of patients with abundant ITIH5 expression remained and achieved statistical significance (log rank *p* = 0.0371 *). The median OS was 38 months in the high ITIH5 group, compared to 19 months in the low ITIH5 group. The risk of death was significantly reduced in the high ITIH5 expression group (HR = 0.5653, 95% CI = 0.3412–0.9368). To investigate whether ITIH5 expression represents an independent predictor of survival, Cox regression analyses were conducted. In univariate regression, ITIH5 demonstrated a statistically significant impact on OS (HR = 0.58; *p* = 0.04 *). This effect persisted through a multivariate Cox regression, without reaching statistical significance, however (HR = 0.61; *p* = 0.10). The results highlight that ITIH5 expression is indeed an independent predictor of survival in CCA patients, even when accounting for possible confounding variables (Tables S5 and S6).

### 3.3. ITIH5 Expression in CCAs in Relation to Perineural Invasion and UICC Tumor Stages

**Figure 3:**
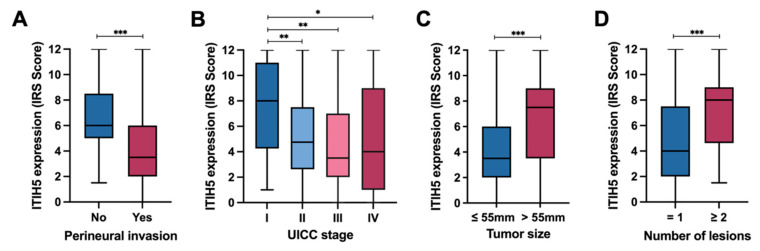
Analysis of the association between ITIH5 protein expression and clinical–pathological parameters. (**A**) ITIH5 expression is notably lower in tumors with perineural invasion; (**B**) Comparison of ITIH5 expression levels between UICC stage I and stages II/III/IV; (**C**) ITIH5 expression is significantly higher in tumors larger than 55 mm; (**D**) Increased ITIH5 expression is observed in cases with more than two lesions. * *p* < 0.05, ** *p* < 0.01, *** *p* < 0.001.

The correlation of ITIH5 expression and the clinical pathological parameters of our CCA cohort applying the Fisher’s Exact Test (Table 1) revealed significant associations between ITIH5 expression and the tumor type, tumor size, number of lesions, vascular invasion, perineural invasion and UICC tumor stage. Tumors with high ITIH5 expression correlate with the diagnosis of iCCA, the absence of perineural invasion and a lower UICC tumor stage. Perineural invasion (PNI) is defined as the invasion of nerves and the perineural area by tumor cells, a mode of tumor spread [40]. As shown in Figure 3A, tumors without PNI were characterized by higher ITIH5 expression (mean IRS of 6.66 in samples without PNI vs. 4.22 in samples with PNI, *p* = 0.0001 ***). We further analyzed the association between ITIH5 expression and the tumor stage. Figure 3B shows a steady decline in ITIH5 expression, with tumor progression represented by the UICC stage. A Mann–Whitney U test revealed a significant difference in ITIH5 expression between stage I and stage II (*p* = 0.0014 **), stage I and stage III (*p* = 0.0016 **) and stage I and stage IV (*p* = 0.0219 *). Interestingly, the results of Figure 3C,D show that large-sized tumors and multifocal tumors have higher ITIH5 expression. This can be attributed to the fact that the majority of tumors in our CCA TMA cohort larger than the median diameter of 55 mm were iCCA (iCCA/tumor = 72/82), whereas most tumors with a diameter of 55 mm or less were pCCA (pCCA/tumor = 65/87). Furthermore, almost all multifocal tumors were iCCA (iCCA/tumor = 31/32), whereas the numbers of iCCA and pCCA were roughly the same for unifocal tumors (pCCA/tumor = 79/143). Typically, iCCA presents with a larger tumor size at diagnosis due to the absence of early symptoms, in contrast to pCCA, which is often detected earlier due to obstructive symptoms [41]. In addition, the multicentric origin, the rich hepatic blood supply and the histological characteristics contribute to the more frequent multifocality in iCCA, while the anatomical location and biological behavior of pCCA generally lead to a unifocal presentation [1].

### 3.4. ITIH5 Re-Expression in Cholangiocarcinoma Cell Lines May Impair Colony Growth but Not Cell Proliferation

**Figure 4:**
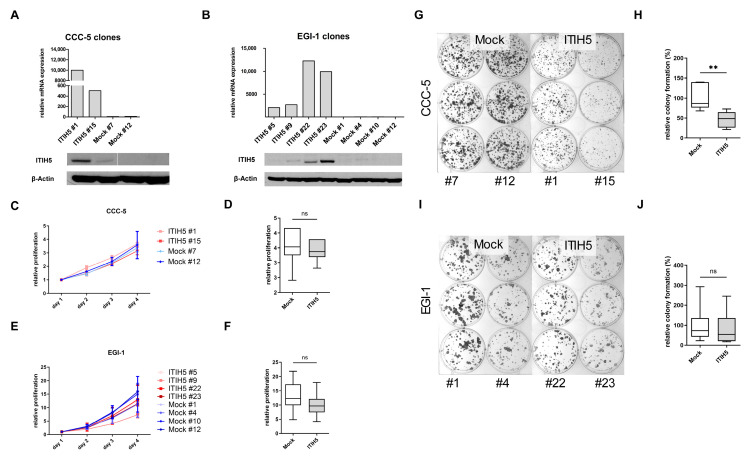
Forced ITIH5 re-expression significantly impairs colony formation of the CCC-5 cell line. (**A**,**B**) Single-cell ITIH5 overexpressing clones of cell lines CCC-5 and EGI-1 was established. Gain-of-function models show significant re-expression of ITIH5 in the ITIH5 clones on mRNA and protein levels in both CCC-5 (#1, #15) and EGI-1 (#5, #9, #22, #23). β-Actin served as a loading control in the Western blot; (**C**–**F**) The XTT assay shows no significant difference in relative proliferation over 96 h between ITIH5 and mock clones. The metabolic activity on day 1 was set as the baseline for each clone; (**G**,**H**) Colony formation assay shows a significantly reduced colony formation in ITIH5-overexpressing CCC-5 clones after 14 days; (**I**,**J**) No significant differences were observed between ITIH5 and mock clones in the colony formation assay in the EGI-1 cell line. ns, not significant, ** *p* < 0.01.

With regard to the prognostic significance of ITIH5 expression in cholangiocarcinoma, we hypothesized that ITIH5 is functionally involved in mechanisms that may inhibit tumor progression in this tumor entity. To functionally test this hypothesis, we restored ITIH5 expression in the two eCCA cell lines, i.e., CCC-5 and EGI-1 by stable transfection with a full-length ITIH5 cDNA pBK-CMV expression vector (ITIH5 clones) or the empty vector alone (mock clones). Ectopic ITIH5 expression in the stable CCC-5 and EGI-1 clones was confirmed by real-time PCR and Western blotting (Figure 4A,B). We then used these *in vitro* tumor models to analyze the effects of ITIH5 re-expression on tumor cell behavior. Although no significant effect on the proliferation rate of CCC-5 and EGI-1 cancer cells was observed (Figure 4C–F), colony formation ability, which to some extent reflects the ability of a tumor to spread, was significantly impaired by ITIH5 in CCC-5 tumor cells (Figure 4G,H). Densitometric analysis revealed a highly significant delay in colony formation in ITIH5-expressing CCC-5 cells by 38.29 % (*p* < 0.01). For EGI-1, the effect did not reach the significance threshold (*p* = 0.37) (Figure 4I,J).

### 3.5. ITIH5 mRNA Expression Correlates with Promoter Hypomethylation in CCAs

**Figure 5:**
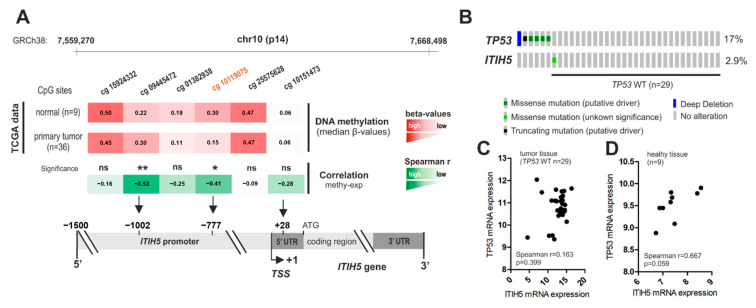
ITIH5 mRNA expression correlates with promoter hypomethylation in CCA, while p53 signaling may lose its potential impact on ITIH5 expression. (**A**) Visualization of the *ITIH5* promoter region, methylation level (mean β-values) of 6 CpG sites in normal and tumor tissue samples (red) and Spearman correlation of DNA methylation and ITIH5 expression for each CpG site in tumor tissue samples (green); Numbers in (**B**) indicate percentage of tumors exhibiting predicted pathological mutations, which is very low for *ITIH5*; Correlation between TP53 and ITIH5 mRNA expression in CCA cases that are wild-type for TP53 (**C**) and healthy tissue (**D**) using the TCGA dataset. ns, not significant, * *p* <0.05, ** *p* < 0.01.

Given the unexpected overexpression of ITIH5, associated with favorable prognosis in CCA, we finally aimed to decipher putative reasons for its gene regulation. Since the *ITIH5* tumor suppressor gene has been shown to be frequently silenced by promoter DNA hypermethylation in various entities [16,21,25], we first focused on its epigenetic configuration and whether an association with mRNA expression could be confirmed. For this purpose, the DNA methylation of six CpG dinucleotides in the *ITIH5* promoter region was analyzed and correlated with ITIH5 mRNA expression using the TCGA CCA dataset. All six CpG dinucleotides are located in the upper region of the *ITIH5* promoter (−1818 bp to +28 bp relative to the expected TSS, Figure 5A), which have already been characterized as diagnostically particularly relevant in an earlier publication of our research group [16]. Interestingly, abundant promoter DNA methylation was present in normal tissue (mean methylation: 0.29), while CpG sites were characterized by hypomethylation in cancerous tissues (mean methylation: 0.257), especially the CpG sites cg09445472 and cg10119075. Consistent with previous findings [25], the hypomethylation of cg10119075 (Spearman’s ρ = −0.46, *p* = 0.001 **) and cg09445472 (Spearman’s ρ = −0.46, *p* = 0.001 **) was significantly associated with increased ITIH5 expression, suggesting a role as molecular mechanisms for *ITIH5* gene regulation by providing an open and accessible promoter region.

Since Liu and colleagues showed the transcriptional activation of the *ITIH5* gene by p53 [42], we next correlated TP53 and ITIH5 gene expression in the CCA TCGA dataset (Figure 5B–D). Samples with pathogenic mutations in the TP53 gene indicating a non-functional p53 signaling were excluded from the Spearman rank coefficient calculations, as an altered p53 protein may not be able to activate ITIH5 transcription [34]. Overall, no positive correlation between TP53 mRNA and ITIH5 mRNA expression was detectable in CCA cases that were wild-type for *TP53*. Interestingly, the expression of both genes tends to be co-regulated in healthy tissue in spite of increased *ITIH5* promoter DNA methylation, arguing for a general p53 responsiveness of *ITIH5* in normal cells, while p53 signaling may lose its impact on ITIH5 expression in CCA.

## 4. Discussion

Patients with CCA are usually diagnosed at advanced tumor stages using conventional methods such as imaging and histological confirmation [12]. In addition, CCA patients have a poor prognosis that is not sufficiently improved by current classic treatment options like surgical resection and chemotherapy [43,44]. A more personalized approach could improve the treatment options and thus the prognosis of CCA. For this purpose, molecular diagnostic and prognostic markers are indispensable [45,46]. Two examples of approved personalized therapy drugs used for CCA therapy are Pemigatinib, which functions as an FGFR2 inhibitor [47], and Ivosidenib, which represents an IDH1 inhibitor [48]. In order to broaden the spectrum of options, the molecular pathways involved in the development of CCA tumors needs to be further explored [49].

Numerous studies in the last 15 years have shown that the well-characterized tumor suppressor gene *ITIH5* is epigenetically silenced, and thus, ITIH5 transcription is considerably downregulated in many tumor entities [16,18,19,22,25,26], while forced re-expression in tumor cell lines has demonstrated strong tumor suppressive effects, like a reduction in proliferation and the migration and invasion of cells [16,19,25]. In stark contrast to these previous observations, we have now defined CCA as a tumor entity, where ITIH5 expression is strongly upregulated in the tumor tissue compared to normal tissue (which is bile duct tissue in the case of CCA). This raises the question of whether ITIH5, although higher expressed, still has a tumor suppressive function, as in all other tumor entities described so far, or whether it may exceptionally act as a cancer-promoting factor in this specific tumor entity. To answer this question, overall survival rates were analyzed, comparing patient groups with low and high ITIH5 expression. Interestingly, the patient group with high ITIH5 expression still demonstrates a favorable overall survival, like in tumor entities such as breast [16] and bladder cancer [19], where ITIH5 is clearly downregulated in the tumor, compared to their normal tissue counterparts. In many cancer entities like, e.g., breast, bladder and pancreatic cancer, it was described previously that *ITIH5* represents a typical class II tumor suppressor gene [50] being silenced by epigenetic promoter hypermethylation [19,26]. Transferring this mechanism to CCA, one might expect an opposite starting position and course of events and thus high *ITIH5* promoter DNA methylation in normal tissue where ITIH5 expression is low (and possibly epigenetically silenced) and a reduced or completely lost *ITIH5* promoter DNA methylation in CCA tumor tissue where ITIH5 mRNA and protein are strongly expressed. So far, we could only analyze this putative relation between DNA methylation and expression in the tumor samples of the TCGA dataset. It would be meaningful to assess DNA methylation in pathological specimens from patients with varying ITIH5 expression levels in the TMA. Indeed, when comparing the mean DNA methylation of six CpG sites in the *ITIH5* promoter between low and high ITIH5-expressing tumors, we observed the expected negative correlation between *ITIH5* promoter DNA methylation and ITIH5 mRNA expression using Spearman correlations. This is consistent with the previous findings that ITIH5 expression is regulated by promoter DNA methylation.

Perineural invasion (PNI) has been described as an independent predictor associated with a poor prognosis in CCA in several studies [51,52]. Interestingly, our data demonstrate a connection between ITIH5 expression and the absence of PNI. Namely, in patients with high ITIH5 expression, PNI is observed significantly less frequently. This may lead to the hypothesis that the prevention of perineural invasion could be a mechanism of ITIH5`s tumor suppressive action in CCA. Furthermore, in the CCA TMA data, a tendency of higher ITIH5 expression in earlier tumor stages was observed. This may suggest an initial upregulation of ITIH5 in the early tumor stages, possibly as a protection mechanism of the organ by taking advantage of its tumor suppressive effects.

Interest in ITIH5 biology in the field of basic cancer research but also personalized diagnostics of cholangiocarcinoma could increase significantly in the future. Recently, it was shown by Chen et al. that serum ITIH5 levels are considerably elevated in cholangiocarcinoma compared to controls (i.e., hepatocellular carcinoma, benign disease, chronic hepatitis B and healthy individuals) [32]. Serum ITIH5 yielded areas under the ROC curve (AUCs) of 0.839 to 0.851 to discriminate cholangiocarcinoma from controls, and the combination of ITIH5 with carbohydrate antigen 19-9 (CA19-9) improved the diagnostic efficacy even further [32]. We have now shown in our study that ITIH5 can also be a prognostic marker in CCA tumor tissue. The clear interplay of ITIH5′s prognostic significance in both the tissue and blood of CCA patients could make ITIH5 an important tool in the personalized medicine of iCCA, similar to other CCA biomarkers that have been discussed recently [53]. In addition, tumor suppressor proteins such as ITIH5 could also become interesting for the cancer therapy of cholangiocarcinoma in the longer term [54]. One possible therapeutic approach is the recently proposed concept of small-molecule tumor mimetics for class II tumor suppressor genes (C2TSGs), which aims to phenotypically mimic the effect of tumor suppressor proteins such as ITIH5 [17].

We are aware that this study has several limitations. Our hypothesis is that ITIH5 expression is initially upregulated by *ITIH5* promoter DNA hypomethylation and potentially later downregulated again during tumor progression. To understand the time course of ITIH5 upregulation in CCA tumor development more comprehensively, additional data on early tumor stages, e.g., in biliary intraepithelial neoplasia (BilIN) [55] and intraductal papillary neoplasm of the bile duct (IPNB) [56], are required. These precursor lesions have to be analyzed in detail by ITIH5 expression and methylation analysis. However, as CCA is a rare disease that is mostly diagnosed in advanced tumor stages, sample collection for early tumor stages is compromised. Our study so far has focused on ITIH5 mRNA and protein data. To gain further insight into the tumor suppressive function of ITIH5 in CCA, a much more comprehensive analysis of CCA *in vitro* models than presented here is necessary. The corresponding author would also be grateful to readers of this article, who could provide additional CCA cell lines as part of a collaboration.

In summary, for the first time, we described an upregulation of ITIH5 expression in cholangiocarcinoma on mRNA and protein levels compared to normal tissue, abundant ITIH5 expression in the tumor still being associated with favorable overall survival.

## 5. Conclusions

In conclusion, ITIH5 shows low expression in normal bile duct tissue and is significantly upregulated in cholangiocarcinoma, especially in intrahepatic CCA. ITIH5 upregulation may be associated with *ITIH5* promoter DNA demethylation. ITIH5 expression in CCA is still associated with favorable overall survival; therefore, ITIH5 has a potential tumor suppressive effect in CCA.

## Figures and Tables

**Table 1:** Clinical pathological parameters of the CCA TMA cohort. * *p* < 0.05, ** *p* < 0.01, *** *p* < 0.001.

Variable		N	ITIH5 Low	ITIH5 High	*p*-Value
		175	137	38	
Gender	Male	93	74	19	0.8946
	Female	80	63	17	
	Unknown	2	-	-	
Age	≤Median (68 y)	92	72	20	0.9933
	>Median (68 y)	83	65	18	
Tumor type	iCCA	93	61	32	<0.0001 ***
	pCCA	79	75	4	
	Mixed type	2	-	-	
	GBC excluded	1	-	-	
Tumor size	≤Median (55 mm)	87	79	8	<0.0001 ***
	>Median (55 mm)	82	52	30	
	Unknown	6	-	-	
Number of lesions	1	143	118	25	0.0041 **
	≥ 2	32	19	13	
Lymph node invasion	Yes	49	41	8	0.2708
	No	112	85	27	
	Unknown	14	-	-	
Vascular invasion	Yes	59	52	7	0.0411 *
	No	107	80	27	
	Unknown	9	-	-	
Perineural invasion	Yes	79	71	8	0.0359 *
	No	31	23	8	
	Unknown	65	-	-	
Liver cirrhosis	Yes	5	5	0	0.5867
	No	170	132	38	
Tumor Grade	G1 + G2	113	88	25	0.3696
	G3 + G4	50	42	8	
	Unknown	12	-	-	
UICC Tumor stage	I	37	23	14	0.0074 **
	II + III + IV	138	114	24	
Resection margin	R0	136	107	29	0.7366
	R1 + R2	23	18	5	
	Unknown	16	-	-	

## Data Availability

The data that support the findings of this study are available from the corresponding author upon reasonable request.

## Supplementary Materials

The following supporting information can be downloaded at: https://www.mdpi.com/article/10.3390/cancers16213647/s1, Figure S1: Mean ITIH5 mRNA expression in normal (bile duct) versus tumor tissue of cholangiocarcinoma according to TCGA data; Figure S2: ITIH5 mRNA expression and DNA methylation in the intrahepatic and extrahepatic cholangiocarcinoma cell lines; Figure S3: Real-time PCR analyses of ITIH5 mRNA expression in extrahepatic cholangiocarcinoma cell lines in untreated cells (control) versus those treated with 5-Aza-2′-Deoxycytidine (AZA), Trichostatin A (TSA) or a combination of AZA/TSA; Figure S4: The original uncropped Western blot images; Table S1: Clinical and pathological parameters of samples in the CCA TMA; Table S2: Clinical and pathological parameters of samples in the TCGA cohort CHOL; Table S3: Real-time PCR primer sequences; Table S4: Primer sequences pyrosequencing; Table S5: Survival data for various clinical characteristics for the CCA TMA; Table S6: Multivariate Cox regression model.
