# Supplementary material for: High Expression of the Tumor Suppressor Protein ITIH5 in Cholangiocarcinomas Correlates with a Favorable Prognosis
Source: Cancers (Basel). 2024 Oct 29;16(21):3647. doi: 10.3390/cancers16213647 (PMC11545166; doi:10.3390/cancers16213647)
Supplement: Supplementary file 1 [file cancers-16-03647-s001.zip › cancers-3156583-supplementary.pdf]

Supplementary Materials

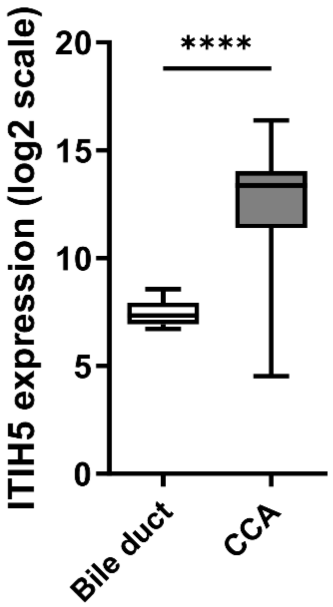

Supplementary Figure S1. Mean ITIH5 mRNA expression in normal (bile duct) versus tumor tissue of cholangiocarcinoma according to TCGA data. Note the logarithmic representation, which corresponds to an approx. 65-fold increase in ITIH5 mRNA expression in CCA tumors compared to normal bile duct tissue. **** p < 0.0001.

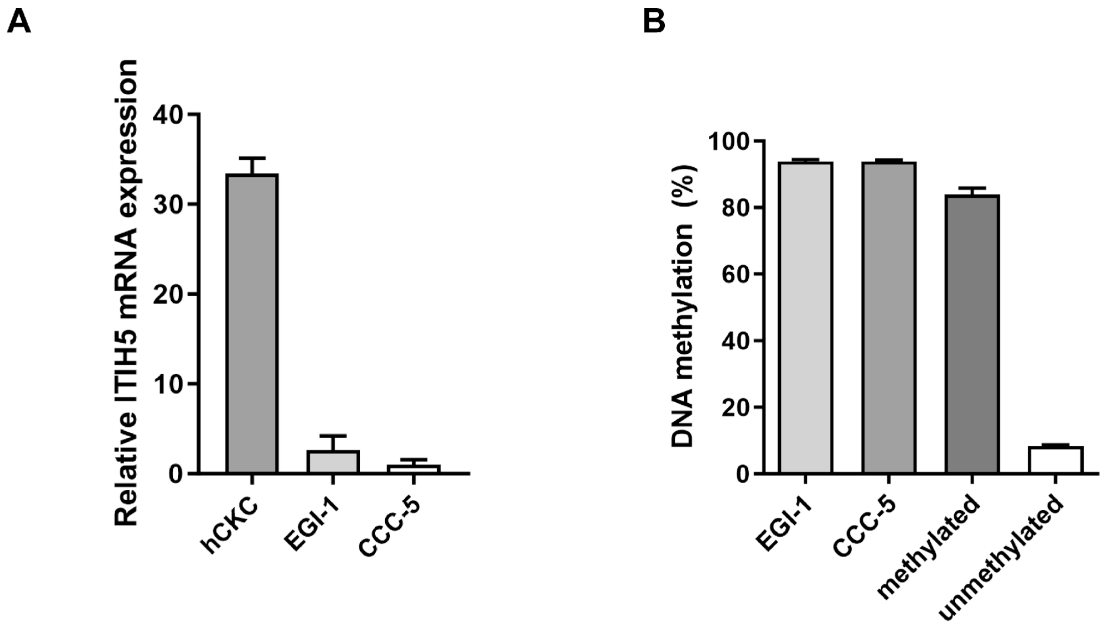

Supplementary Figure S2. ITIH5 mRNA expression and DNA promoter methylation in intrahepatic and extrahepatic cholangiocarcinoma cell lines. (A) Real-time PCR analyses of ITIH5 mRNA expression in the hCKC (intrahepatic cholangiocarcinoma cell line), EGI-1 and CCC-5 (extrahepatic cholangiocarcinoma cell lines). ITIH5 is relatively abundantly expressed in the hCKC cell line. (B) *ITIH5* promoter DNA methylation analysis by pyrosequencing of the EGI-1 and CCC-5 cholangiocarcinoma cell lines. In both cell lines, the *ITIH5* promoter is strongly methylated. Unmethylated and methylated controls are shown as well.

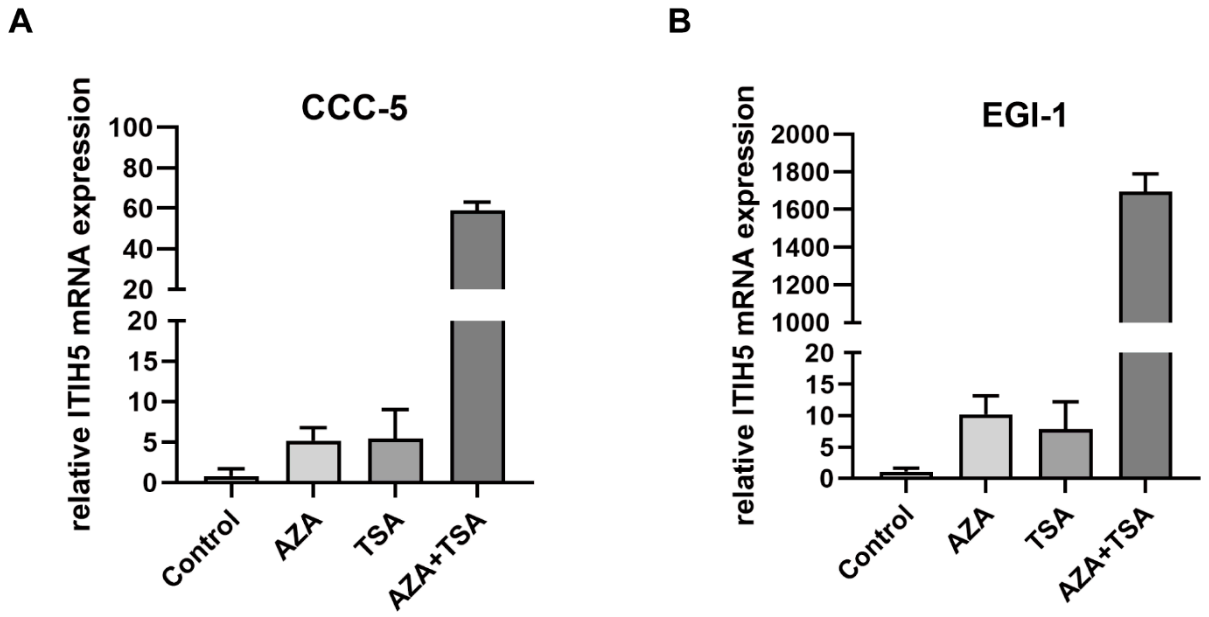

Supplementary Figure S3. Real-time PCR analyses of ITIH5 mRNA expression in extrahepatic cholangiocarcinoma cell lines in untreated cells (control) versus those treated with 5-Aza-2´-Deoxycytidine (AZA), Trichostatin A (TSA) or a combination of AZA/TSA. AZA and TSA were used for demethylation and deacetylation, respectively. (A) In the CCC-5 cell line treatment with AZA or TSA alone resulted in an approximately 5-fold change in ITIH5 mRNA expression, compared to the untreated control group, whereas the combined AZA+TSA treatment induced an upregulation exceeding 50-fold. (B) In the EGI-1 cell line treatment with AZA or TSA alone resulted in an approximately 10-fold upregulation of ITIH5 mRNA expression compared to the untreated control group, whereas the combined AZA+TSA treatment induced a strong upregulation exceeding 1500-fold. Thus, in both cell lines, the combination of AZA+TSA resulted in a much higher ITIH5 mRNA re-expression.

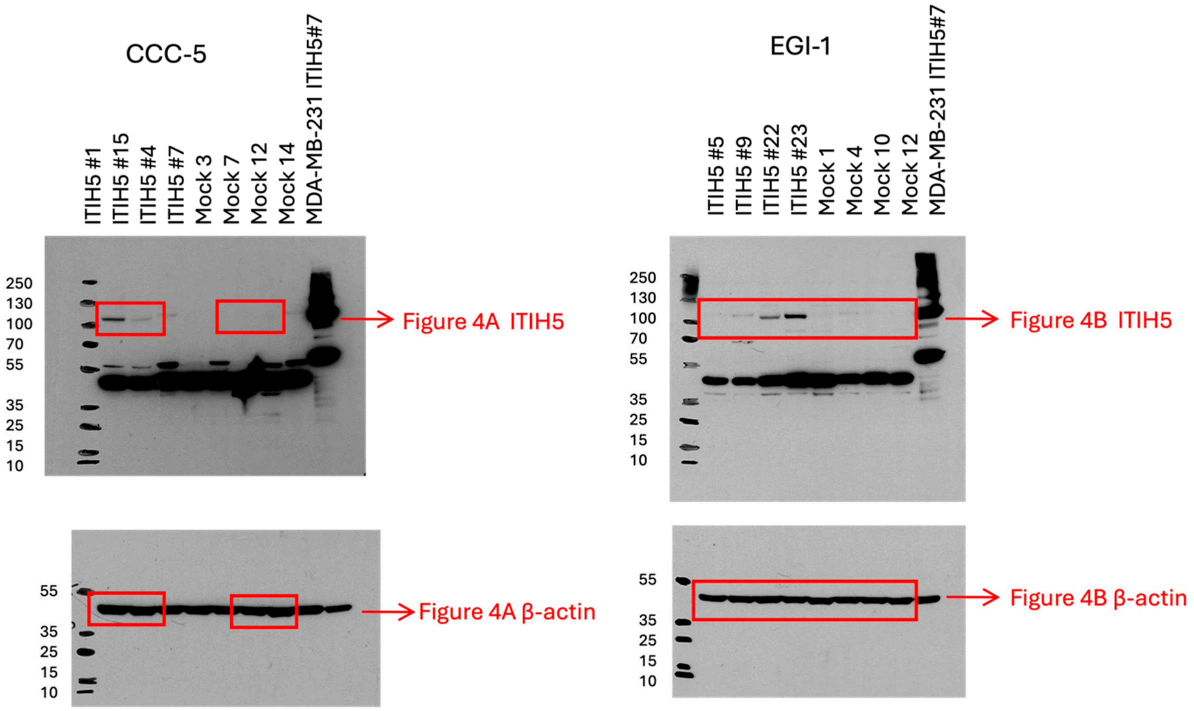

Supplementary Figure S4. The original uncropped Western blot images. The blots marked by the red rectangle are the target proteins.

Supplementary Table S1. Clinical and pathological parameters of samples on the CCA TMA.

**Items**	**Variable**	**Number**
Tissue type	Tumor tissue<br>Normal tissue	175<br>100
Tumor type	Intrahepatic CCC<br>Perihilar CCC (extrahepatic)<br>Mixed type<br>GBC excluded	93<br>79<br>2<br>1
Age (years)	Median	68
Gender	Male<br>Female<br>Unknown	93<br>80<br>2
Overall survival status	Deceased<br>Living/Censored	118<br>57
Overall survival (months)	Median	19
UICC tumor stage	I<br>II<br>III<br>IV	37<br>92<br>31<br>15
Perineural invasion	Yes<br>No<br>Unknown	79<br>31<br>65

Supplementary Table S2. Clinical and pathological parameters of samples in the TCGA cohort CHOL.

**Items**	**Variable**	**Number**
Tissue type	Tumor tissue<br>Normal tissue	36<br>9
Tumor type	Intrahepatic CCC<br>Extrahepatic CCC	30<br>6
Age (years)	Median	66.5
Gender	Male<br>Female	16<br>20
Overall survival status	Deceased<br>Living/Censored	18<br>18
Overall survival (days)	Median	645
UICC Tumor stage	I<br>II<br>III<br>IV	19<br>9<br>1<br>7
Perineural invasion	Yes<br>No	7<br>26

Supplementary Table S3. Real-time PCR primer sequences.

Gene		Primer sequence 5' --> 3'	Annealing temperature	Amplicon size
ITIH5_202	Forward<br>Reverse	TTCCTCACTCCCTTCACCTC<br>TGGCTTCTTGAGCAAAGGTC	60°C	159 bp
GAPDH	Forward<br>Reverse	GAAGGTGAAGGTCGGAGTCA<br>AATGAAGGGGTCATTGATGG	60°C	108 bp

Supplementary Table S4. Primer sequences for pyrosequencing.

Gene		Primer sequence 5' --> 3'	Amplicon size
ITIH5	Forward<br>Reverse<br>Sequencing	AGGGGTTYGTGGGGTTAATATAGGTGGTTT<br>RCCRCTTCCCRACCTCAATCCC<br>GGGGTTAATATAGGTGGTT	138 bp

Supplementary Table S5. Survival data for various clinical characteristics for the CCA TMA. Hazard ratios (HR) calculated using a univariate Cox regression model.

Factor	N	Median OS, months	HR (95% CI)	P
**Tissue type**				
Normal tissue	100	-		
Tumor	175	19		
**Gender**				
Male	93	16	1.09 (0.74 – 1.61)	
Female	80	22	0.92 (0.62 – 1.36)	0.67
Unknown	2	-	-	-
**Age**				
≤ Median (68 y)	92	19	1.11 (0.75 – 1.65)	0.59
> Median (68 y)	83	18	0.90 (0.61 – 1.33)	
**Klatskin tumor**				
Yes	78	17	0.72 (0.48 – 1.09)	0.12
No	97	20	-	-
**Tumor type**				
Intrahepatic CCC	93	20	1.33 (0.89 – 1.98)	0.16
Perihilar CCC	79	17	0.72 (0.48 – 1.09)	0.12
Mixed type	2	26	-	-
GBC excluded	1	0	-	-
**Cirrhosis**				
Yes	5	5	1.60 (0.50 – 5.05)	0.43
No	170	19	-	-
**Tumor size**				
≤ Median (55 mm)	87	20	0.55 (0.37 – 0.82)	**0.004 ****
> Median (55 mm)	82	14.5	1.82 (1.21 – 2.72)	
Unknown	6	-	-	-
**Number of lesions**				
1	143	19	0.53 (0.34 – 0.83)	**0.006 ****
≥ 2	32	16.5	1.74 (1.14 – 2.65)	
**ITIH5 expression**				
Low	137	17	1.70 (1.02 – 2.84)	**0.04 ***
High	38	27.5	0.58 (0.35 – 0.98)	

**Tumor grade**			2.49 (1.75 – 3.56)	**0.001** **
G1	1	43	-	-
G2	112	25	-	-
G3	47	6	-	-
G4	2	7.5	-	-
Unknown	13	-		
**UICC tumor stage**			1.57 (1.23 – 2.00)	**0.001** **
I	37	36	-	-
II	92	20	-	-
III	31	13	-	-
IV	15	6	-	-
**Lymph node invasion**				
Yes	49	11	2.02 (1.31 – 3.13)	**0.001** **
No	112	25	-	-
Unknown	14	-	-	-
**Vascular invasion**				
Yes	59	18	1.63 (1.09 – 2.43)	**0.02** *
No	107	20	-	-
Unknown	9	-	-	-
**Perineural invasion**				
Yes	79	13	1.20 (0.71 – 2.05)	0.49
No	31	18	-	-
Unknown	65	-	-	-
**Resection margin**				
R0	136	19.5	1.46 (0.93 – 2.30)	0.10
R1	22	6.5	-	-
R2	1	71	-	-
Unknown	16	-	-	-

Supplementary Table S6. Multivariate Cox regression model. Only independent risk factors are shown. Included in the analysis were all factors from Supplementary Table S5 with a P-value ≤ 0.05.

**Factor**	**HR**	**P**
Tumor size ≤ Median	0.57	**0.03 ***
Lymph node invasion	1.87	**0.02 ***
High ITIH5 expression	0.61	0.10
